# Red Sea Opisthobranchia 5: new species and new records of chromodorids from the Red Sea (Heterobranchia, Nudibranchia, Chromodorididae)

**DOI:** 10.3897/zookeys.770.26378

**Published:** 2018-07-04

**Authors:** Nathalie Yonow

**Affiliations:** 1 Swansea Ecology Research Team, Department of Biosciences, Swansea University, Singleton Park, Swansea SA2 8PP, Wales, United Kingdom

**Keywords:** Biogeography, nomenclature, sea slug, taxonomy, western Indian Ocean

## Abstract

This is the fifth publication describing species of sea slug heterobranchs, originally based on collections from the Red Sea by the author on four expeditions carried out in 1983 and 1990, with the addition of specimens subsequently collected by underwater photographers who were stimulated by the book "Sea Slugs of the Red Sea". So much material has been amassed that only the new species and new Red Sea records of chromodorids are described in this paper, with an appendix listing specimens of previously recorded species. Three new species are described in detail and illustrated, belonging to three different genera: *Doriprismatica kyanomarginata* **sp. n**., *Glossodoris kahlbrocki* **sp. n.**, and *Goniobranchus pseudodecorus* **sp. n.** One western Pacific species is recorded for the first time in the Red Sea, *Goniobranchus collingwoodi* (Rudman, 1987). The nomenclature of *Verconia sudanica* is discussed and stabilised.

## Introduction

This series of papers is based on the author’s collections in the Red Sea during four expeditions in 1983 and 1990. Subsequent material was provided by underwater photographers and scientists who collected in the Egyptian Red Sea. The first two papers based on these collections dealt with the families Phyllidiidae (Yonow 1988) and Chromodorididae (Yonow 1989), while the third dealt with the families Polyceridae, Gymnodorididae, Discodorididae, and Hexabranchidae as well as a few Sacoglossa and Aglajidae (Yonow 1990). A fourth publication described some non-nudibranch sea slugs, the Cephalaspidea, Anaspidea, and Pleurobranchida, as well as the dendronotid and aeolid nudibranchs (Yonow 2000). The book "Sea Slugs of the Red Sea" (Yonow 2008) provided some further photographic evidence (some with specimens) of described and undescribed species, as well as a detailed checklist of species with all records (pp 59–61 for chromodorids), and a section entitled *Incertae Sedis* with photographs of unidentified chromodorids (p 275). Finally, new records and a new species are illustrated in Yonow (2015).

This paper deals with a number of specimens collected by the author but also includes specimens collected more recently by Johann Hinterkircher, Sven Kahlbrock, Ernesto Mollo, and Ángel Valdés in the northern Red Sea. One species each of the genera *Chromodoris*, *Diversidoris*, *Doriprismatica*, *Glossodoris*, *Hypselodoris*, *Miamira*, and *Verconia*, and two species of *Goniobranchus* are described. All species are illustrated with colour figures of living specimens, and any literature relating to the species in question is included in the synonymy for each species, focusing on the Red Sea, Arabian Sea, Persian Gulf, and the wider north-western Indian Ocean.

An appendix lists the additional material pertaining to species already described in the previous papers by this author on the Red Sea, with additional notes and illustrations.

## Materials and methods

The materials and methods employed in the field (for the author’s own collections) and in the laboratory have been described previously and are not repeated here (Yonow 1989, 1998, 1990, 2000). The material collected by Sven Kahlbrock and Johann Hinterkircher was not always measured or relaxed before preservation, but it was almost always accompanied by series of colour photographs. As soon as the material arrived, it was processed in the laboratory by microscope examination, and measurements, notes, and drawings were made of most preserved specimens; specimens were then placed in vials of 70% alcohol with labels. In the ‘Material’ section of each species and in the appendix, specimen refers to a collected animal which has been preserved, registered, and lodged in the Senckenberg Museum, Frankfurt, Germany, while individual refers to an animal which was photographed, some measured alive, but not collected or preserved.

The images of preserved specimens, or their parts, were taken with a Kodak M530 camera and/or an Olympus BX40F4 dissecting microscope. The buccal mass of each specimen was extracted and processed in 10% sodium hypochlorite solution for 1–2 minutes to dissolve connective and muscle tissue, leaving only the radula and the jaws. The features of the radulae and jaws of each species were analysed under the stereomicroscope and scanning electron microscope (JSM). Specimens, SEM stubs, colour slides, and digital images of the material included in this paper will be deposited in the Senckenberg Museum.

## Species accounts

### Chromodoris strigata

Taxon classificationAnimaliaNudibranchiaChromodorididae

Rudman, 1982

Plates 1 , 2

Chromodoris strigata Rudman, 1982: 229–231, figs 17E, 26, 27 (Queensland, Australia; Madagascar); Yonow 2008: 60, 177 (Gulf of Eilat, Red Sea); Tibiriçá et al. 2017: 20, fig. 4C (Mozambique).

#### Material.

Al Fanadir, near Hurghada, Egypt, 26 May 2009, two specimens 16 and 11 mm (preserved), leg. and photograph S Kahlbrock; numerous photographs from northern Egypt, S Kahlbrock and J Hinterkircher; numerous photographs from the Creek, Jeddah, Saudi Arabia, 1970–1994, W Pridgen.

#### Description.

**Plate 1.**
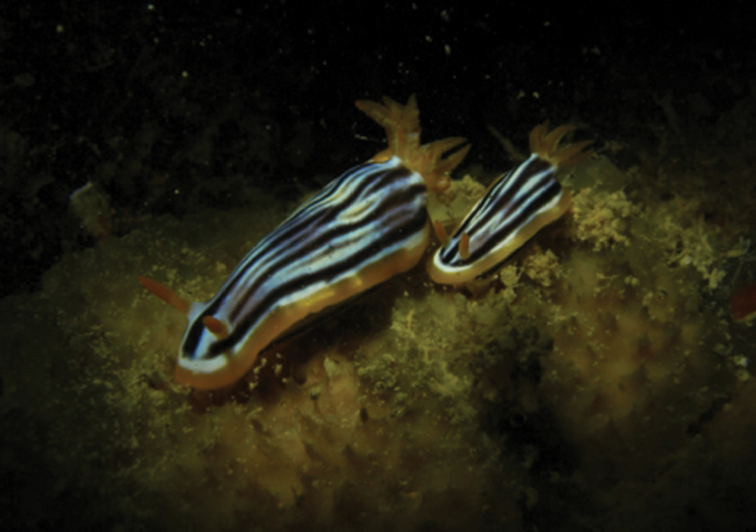
*Chromodoris strigata* Rudman, 1982, photograph S Kahlbrock.

Photographs of the two specimens depict the typical pattern of this species in the Red Sea (Plate 1): there are five black lines on the dorsum, which are broken in the larger specimen. These have the characteristic blurring behind the rhinophores, mid-body, and in front of the gills where the white in-between the black is darker. The middle black line runs anteriorly between the rhinophores. There is a submarginal white band completely encircling the notum followed by a thicker yellow-orange margin. The dorsal surface of the foot is white with an orange margin and two black lines that do not meet on the tip of the tail. The rhinophores are either the same colour as the mantle margin or more orange. The 7–9 gills are the same colour as the rhinophores and bear white pinnules.

The body is elongate and the mantle is raised just in front of the gills. The foot is long and pointed, nearly 1/3 to 1/4 longer than the body length. The rhinophores are long and pointed, usually held out over the sides of the body in a characteristic manner. The gills are simply pinnate, arranged in a circle that is not closed posteriorly; the last gills are smaller than the others.

#### Distribution.

**Plate 2.**
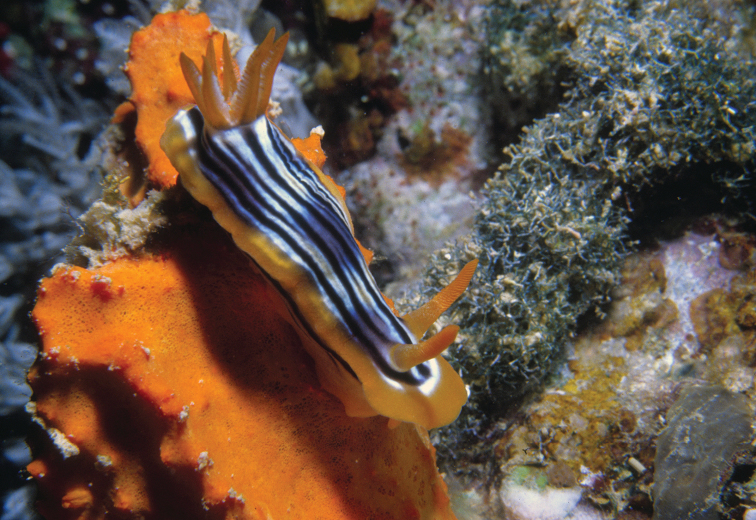
*Chromodoris strigata* Rudman, 1982, photograph E Pridgen.

These are the first specimen records from the Red Sea but an individual had been photographed in the Jeddah area of the Red Sea as early as the 1970’s (W Pridgen pers. comm., Plate 2). There are subsequent records from the northern Red Sea (Yonow 2008) although it was never collected by the author. *Chromodoris strigata* is a western Pacific species with one record in the Indian Ocean, from Mozambique (Tibiriçá et al. 2017). The records from India as *C. strigata* (and *C. colemani*, Sreeraj et al. 2012) are most likely C. cf. hamiltoni Rudman, 1977 as are the records from Mozambique of *Chromodoris* sp. 1 (Tibiriçá et al. 2017).

### Diversidoris flava

Taxon classificationAnimaliaNudibranchiaChromodorididae

(Eliot, 1904)

Plate 3

Chromodoris flava Eliot, 1904: 399 (Zanzibar).
Noumea flava – Rudman 1986c: 379, figs 1–4, 17 (GBR, Australia); Yonow 2008: 61, 199 (Red Sea).

#### Material.

Sha’ab steel tank, Hurghada, Egypt, 01 Aug 2009, 35 m depth on sand, one specimen 4.5 × 2.5 mm (preserved), leg. and photographs S Kahlbrock; photographs only, vicinity of Hurghada, Egypt, 08 Nov 2013, 13 July 2015, S Kahlbrock.

#### Description.

**Plate 3.**
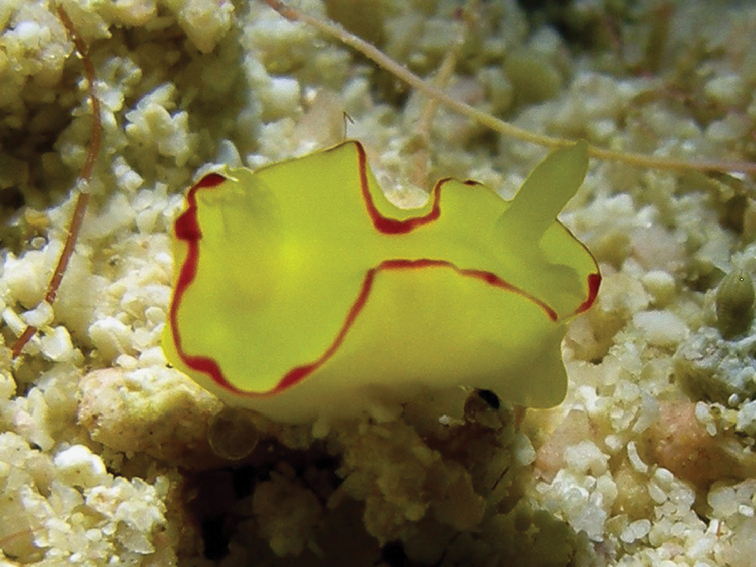
*Diversidoris flava* (Eliot, 1904), photograph S Kahlbrock.

This species is unmistakable with its lemon yellow body bordered by a deep red line along the margin (Plate 3). The rhinophores and unipinnate gills are also lemon yellow. The tiny specimen was damaged, and missing most of its right rhinophore. The left rhinophore bears 12 lamellae, the edges of which are opaque white in life.

The mantle margin of the preserved specimen is of uniform thickness, as is its edge despite the implications of the red line along the margin in life, which is thicker at intervals in the photographs.

#### Remarks.

*Diversidoris flava* was originally described in *Noumea* but it has been shown by Johnson and Gosliner (2012) that it belongs to the genus *Diversidoris*, separated from *Noumea* (= *Verconia*; see Remarks below for *Verconia sudanica*).

#### Distribution.

This is the first specimen record of *Diversidoris flava* in the Red Sea; it was previously recorded by a series of photographs also from Eilat in the northern Red Sea no earlier than 2005 (Eilat, Yonow 2008: 199; http://www.seaslugforum.net/find/21083). The distribution of this somewhat uncommon species is throughout the Indo-Pacific Ocean; its recent arrival in the Red Sea may be due to shipping.

### Doriprismatica kyanomarginata sp. n.

Taxon classificationAnimaliaNudibranchiaChromodorididae

http://zoobank.org/3BA982AA-5F86-4089-8D8B-9534A07F6284

Figure 1 , Plate 4

Colourful sea slugs – Dipper and Woodward 1989: 58.
Glossodoris cincta – Yonow 2008: 60, 188 (lower left large and upper small photographs only, Egypt) (**non**Casella cincta Bergh, 1888).

#### Type material.

**HOLOTYPE SMF 349566**: Egypt, Sept/Oct 1995, one specimen 21 × 13 mm preserved (still retains dorsal mottling, marginal bands clearly broad ochre, light blue line, black margin on both sides), leg. Á Valdés & E Mollo (HU-M7), radula already dissected, used for SEM.

#### Other material.

**Plate 4.**
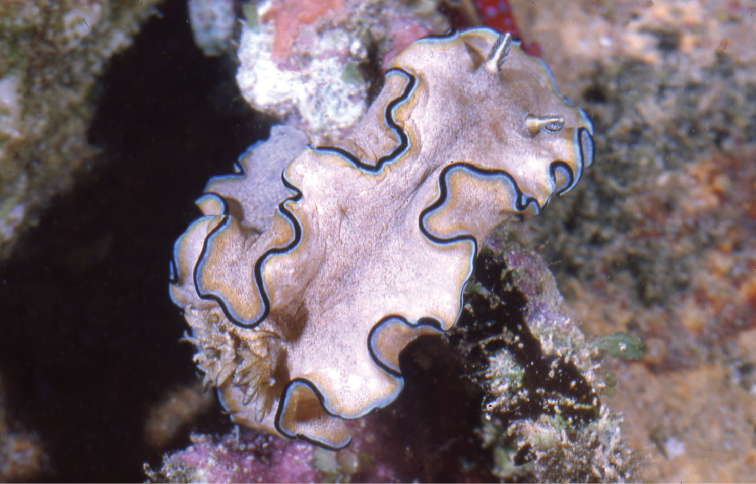
*Doriprismatica kyanomarginata* sp. n., photograph G Brown (non-type).

The Creek, Jeddah, Saudi Arabia, 1980s, photographs of one individual, J Kuchinke (Yonow 2008: 189, uppermost small photograph on right); one individual, photograph G Brown (Plate 4); Egypt, 1990s, photograph of one individual, J Hinterkircher; Egypt, 28 Sept 1995, intertidal, photograph of one individual, Á Valdés & E Mollo (HU-015).

#### Diagnosis.

While the body shape and colour are similar to those of *Glossodoris cincta*, the marginal banding is diagnostic: the diffuse yellow innermost band has a sharp outer line bordering the distinct sky blue band, which is followed by a pitch-black margin visible on both sides of the mantle edge.

#### Description.

This species is distinctive with its fleshy body thrown into four primary and multiple secondary folds. The approximately 20 gills are simply pinnate but whorled around the anal papilla. The rhinophores issue from low raised sheaths, which may bear tiny white spots around the margin, and carry in the region of 21 lamellae.

The body is cream with beige irregularities; this may be somewhat darker centrally in some individuals (Plate 4). The banding on the margin is distinctive: the creamy beige dorsum abruptly becomes more yellow, producing a band that is diffuse on the inside but abruptly demarcated on the outside. It is followed by a light blue line and the mantle margin is marked by a thick black line. This colour pattern is also present on the hyponotum. The gills have a beige-brown line up both sides, which meets over the top of each gill. The pinnules are opaque white. The rhinophores have a mottled beige stalk and the lamellate club is rather rounded. There is a white line up both sides and along the edges of the lamellae.

**Figure 1.**
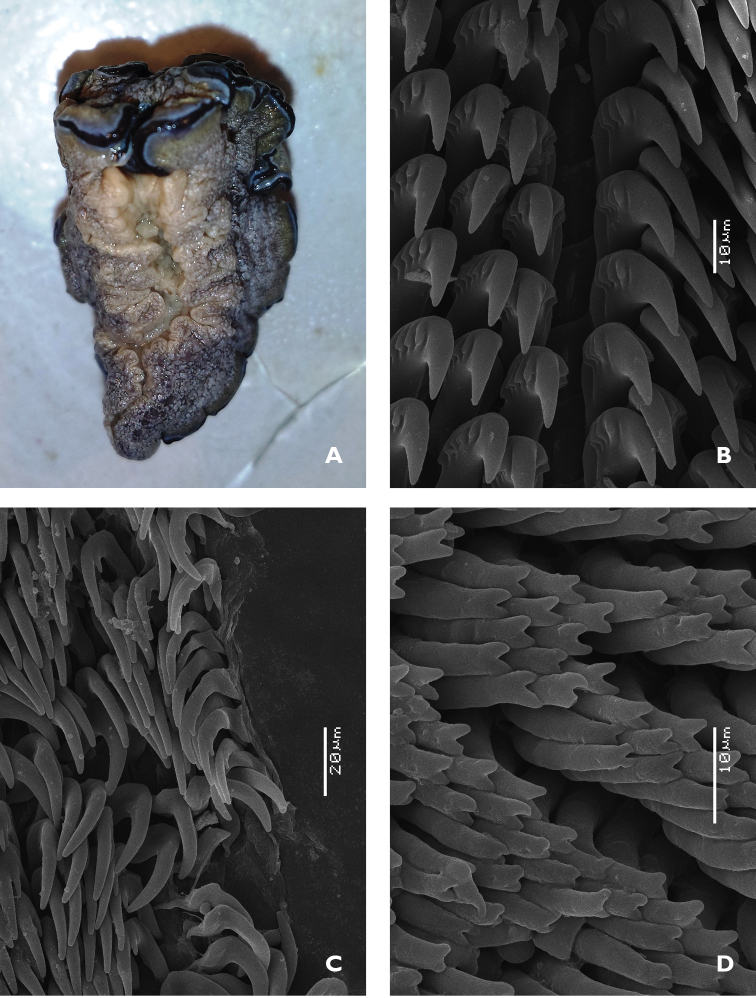
*Doriprismatica kyanomarginata* sp. n. **A** ventral view of whole specimen showing colours of the preserved specimen, including the anterior section of the mantle margin **B** midline area from the anterior portion of the radula **C** lateral teeth from the posterior section of the radula **D** jaw rodlets.

The coloured banding remains on the preserved specimens (Figure 1A). Ventrally, it is creamy beige, with the top of the foot a little darker but not as dark as the hyponotum. There are no coloured bands on the margin of the foot. The anterior margin of the foot is thickened, and the radula was already dissected upon reception.

The radular formula is >73 × approx. 50.0.50. There is no median thickening or rhachidian tooth present, but a small space in the middle (Figure 1B). The first marginal tooth on each side bears four or five denticles on each side of the cusp. The cusp becomes much longer at approximately tooth 4 or 5, and the four or five denticles also become a little larger. The outermost teeth in the row are undifferentiated but somewhat reduced in cusp and root sizes; the denticles are also reduced in both size and number (Figure 1C).

The jaw rodlets are curved and bicuspid at the tips, 20–25 µm long (Figure 1D).

#### Remarks.

Originally considered a colour form of *Glossodoris cincta* by Rudman (1986a) and Yonow (2008), this species is rarely recorded in the Red Sea compared to the common reddish *G. cincta* with the ochre-black-white mantle margin: in fact, there are only four photographic records of this new species from Jeddah and Egypt since the 1970s (see Material above) compared to the many others of *G. cincta*. There are also no records of *D. kyanomarginata* sp. n. from the Red Sea on SeaSlugForum (http://www.seaslugforum.net/showall/gloscinc) nor the internet. There is, however, one photograph of a pair of this new species in a book on the Persian Gulf (Dipper and Woodward 1989) but there are no further records from the Gulf.

The Indian Ocean form of *Glossodoris cincta* is similar to this species, but has a darker body with a similar fading towards the margin and only two coloured marginal bands, a bright yellow submarginal line and a black marginal line: there is no blue. Additionally, the radular and jaw elements differ substantially. There are many more denticles on the teeth of the western Indian Ocean specimen, approximately 8–13 compared to the 4–5 present on the teeth of this new species. The formula of a 55 mm living specimens is 134 × 64.1.64 with a distinct median thickening (Rudman 1986a: 149). The jaws of *Glossodoris cincta* also differ, with most being unicuspid; in *G. kyanomarginata* sp. n. the rodlets are bifid without exception, similar to those of the *G. atromarginata* group of species as defined in Rudman (1986a) but currently attributed to *Doriprismatica* (MolluscaBase 2018).

The body shapes differ from both *Doriprismatica atromarginata* (Cuvier, 1804) and *D. plumbea* (Pagenstecher, 1877) as illustrated by Rudman (1986a) and Yonow (1989) (both as *Glossodoris*) in being much more convoluted with secondary undulations. This is relevant, as the marginal banding of *D. plumbea* is similar in colour, and can often be blue. However, the body colour is much more yellow and darker in *D. plumbea*: Gohar and Abul-Ela (1959) described *D. plumbea* (as *Casella atromarginata*) in detail and subsequently Gohar and Soliman (1967) described the Indian Ocean form of *Glossodoris cincta* (as *Casella obsoleta*) and its development from the Egyptian Red Sea, comparing the two species’ very different modes of development. Clearly, there are morphological, radular, and developmental differences between the two genera.

#### Distribution.

Possibly endemic to the Red Sea. Only one unconfirmed published record from the Persian Gulf.

#### Derivatio nominis.

The specific epithet is built by combining the Greek *κυανός* and Latin *marginata*, referring to the cerulean blue submargin.

### Glossodoris kahlbrocki sp. n.

Taxon classificationAnimaliaNudibranchiaChromodorididae

http://zoobank.org/F69F9509-8562-4FA8-AF72-19D7DADB173E

Figure 2 , Plate 5

Glossodoris sp. 10 Debelius and Kuiter 2007: 189 (El Quseir, Egyptian Red Sea).
Glossodoris sp. 6 Gosliner et al. 2008: 238 (Red Sea).
Glossodoris sp. nov. Yonow 2015: fig. 540, fig. 21 (holotype; Hurghada, Red Sea).

#### Type material.

**HOLOTYPE SMF 349567**: Dahara Wadi Gimal, near Hurghada, Egypt, 18 May 2010, 13 m depth, one specimen 25 × 10 mm preserved, leg. and photographs S Kahlbrock. **PARATYPE SMF 349568**: Dahara Wadi Gimal, near Hurghada, Egypt, 10 Jul 2012, 10 m depth, one specimen approx. 40 mm alive (27 × 10 mm preserved, bent), leg. and photographs S Kahlbrock (SK # 6). **PARATYPE SMF 349569**: Dahara Wadi Gimal, near Hurghada, Egypt, 13 Oct 2016, 12 m depth on rock during night dive, one spcm 15 × 9 mm preserved, leg. and photographs S Kahlbrock (SK # 3; radular and jaw preparations).

#### Other material.

**Plate 5.**
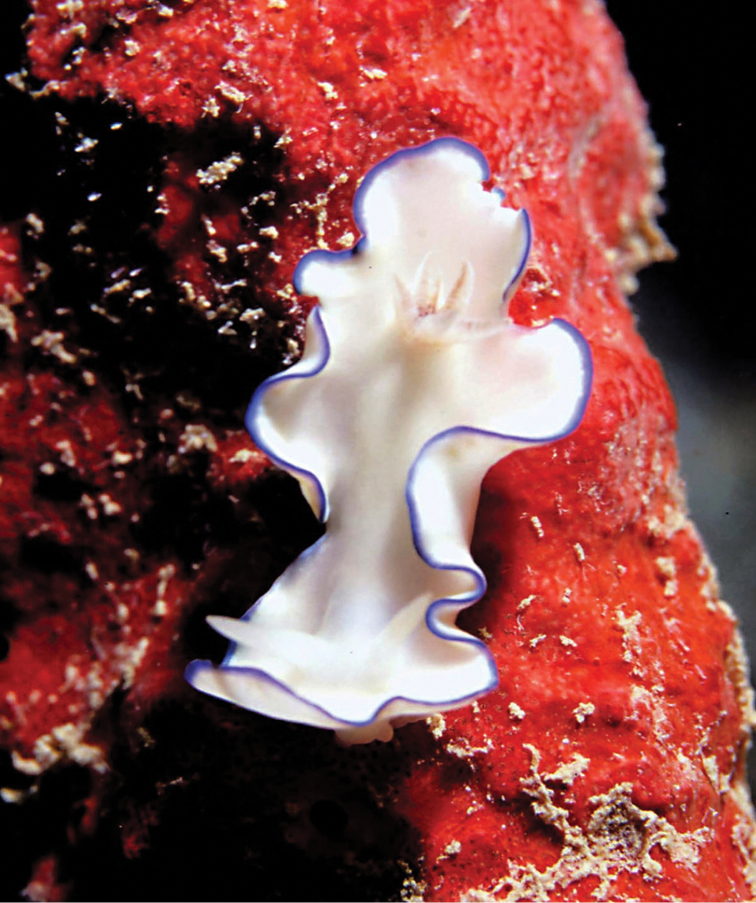
*Glossodoris kahlbrocki* sp. n., photograph E Pridgen (non-type).

The Creek, Jeddah, Saudi Arabia, 1970s, photographs of one individual only, W Pridgen (Plate 5).

#### Diagnosis.

Uniformly white to cream mantle with no markings. Mantle colour bleeding into a more opaque white submargin. Bright blue border same thickness as opaque white band with clear distinct boundaries on both sides. Thin marginal line deep blue to black, present on both dorsal and ventral surfaces. Gills and rhinophores white, gill lamellae may tend to ochre.

#### Description.

This distinctive glossodorid is essentially white with a bright blue margin. The marginal pigmentation is identical in all three specimens (and the few available photographs, see Material above), and present on both sides of the mantle margin: an opaque creamy white band is followed by a light blue band and a deep blue to black marginal line. The gills are retracted into a small pocket in all but one photograph: in only one photograph of a series of photographs of the paratype specimen, the nine gills are extended: they are unipinnate with white rachides and ochre lamellae; in Plate 5 they are also extended and number at least five. They are arranged in a circlet and the last two gills are smallest. The rhinophores are tall and parallel-sided, white with faintly ochre lamellae: there are 21 lamellae in the paratype and 18 lamellae can be counted on the photograph of the 2016 non-type specimen. The rim of the rhinophore pocket is barely raised above the notum.

The body is solid and the thick mantle margin is held in three permanent folds. The hyponotum and top of the foot are identical in colour to the mantle, and there is no colour what-so-ever on the foot margin or oral tentacles.

**Figure 2.**
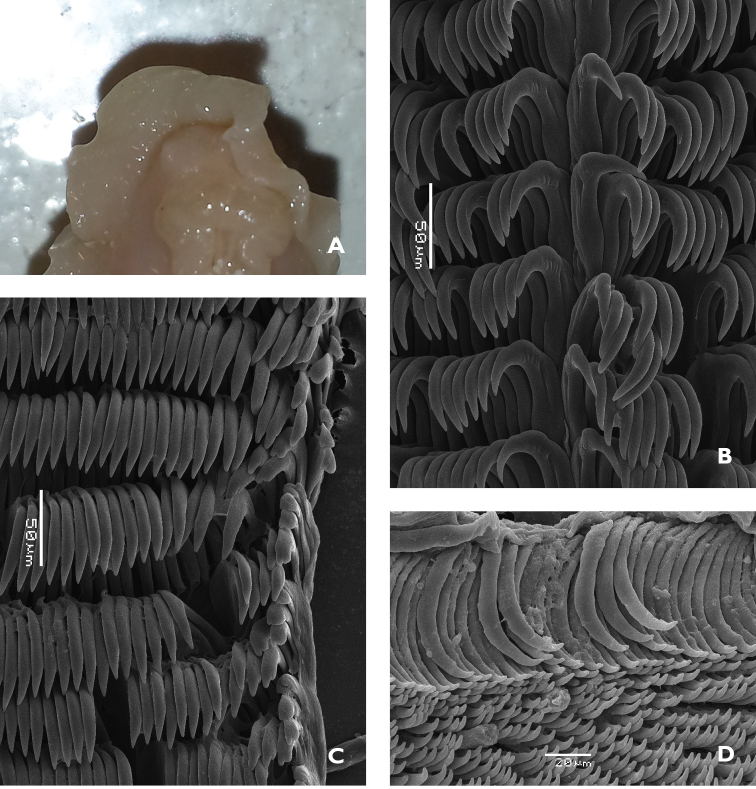
*Glossodoris kahlbrocki* sp. n. **A** ventral view of anterior showing head, oral tentacles, and foot margin **B** midline area from the posterior portion of the radula **C** lateral teeth from the middle section of the radula **D** jaw rodlets.

The preserved holotype is fairly well relaxed and soft. It is elongated and slightly tapered at each end. The dorsum is of almost equal width and height. The mantle margin is very thin and flexible, with the permanent folds visible in the photographs present only as undulations. The foot is much longer than the mantle, and the posterior end is curled over the dorsum. The gill and rhinophore pockets are visible only as puckered holes. Viewed dorsally, the notum is pale pinkish white, the mantle margin is translucent cream. The digestive gland is visible as a dark patch halfway along the body to the left. In ventral view, it is visible as a large sphere spanning the width and depth of the body, therefore visible both dorsally and ventrally. The anterior margin of the foot is rounded and bilaminate; neither lamina is notched. The oral tentacles are two simple swellings each with a terminal nipple (Figure 2A). The type specimens are identical in their preserved states, but the smallest third specimen has a proportionately larger mantle margin. There is no hint of the blue margins on any of the preserved specimens except where the mantle was folded over in the paratype. Mantle glands are visible in a submarginal band on the posterior half of the mantle of the paratype (which also has the front of the foot and head partly damaged).

The reproductive organs of the small 15 mm preserved specimen dissected for the radula preparation were in a relatively underdeveloped state. This is not unexpected as the type specimens are twice the size.

The radular formula of the small specimen is 55 × ~60.0.~60. There is no central tooth in the row; the first tooth in each row bears five or six small rounded denticles on the inner face of the curved cusp (Figure 2B). The remaining teeth have a straight root, longer than the cusp, and a small projection on the top. The teeth are the same shape and dimensions along the row until the last four or five, where they become very reduced in size and stacked in a line (Figure 2C).

The jaws comprise curved rodlets that are conical at the tips, which taper abruptly. They are relatively long, nearly 80 µm in length (Figure 2D).

#### Remarks.

There is absolutely no species of chromodorid resembling this new species, in either the Red Sea or the western Indian Ocean: the pure white dorsum with startling blue marginal bands is unique.

#### Distribution.

Endemic to the Red Sea. The first photograph of this species was taken in the 1970s, in the vicinity of Jeddah, Saudi Arabia (see Material above). Since then, it has only been photographed a few times, indicating its rarity in the Red Sea: the collected specimens are from the same locality years apart. Despite the numerous books and websites on nudibranchs, there are no records of this distinctive species anywhere else in the world.

#### Derivatio nominis.

This species is in honour of Sven Kahlbrock, who searched many years for specimens of this beautiful but rare species. In addition, he has tirelessly supplied photographic records and many specimens in the last eight years.

### Goniobranchus collingwoodi

Taxon classificationAnimaliaNudibranchiaChromodorididae

(Rudman, 1987)

Figure 3 , Plate 6

Chromodoris collingwoodi Rudman, 1987: 358–364, figs 23E-F, 32–35 (eastern Australia, Solomon Islands, Hong Kong); Baba 1989: 23, fig. 2; Rudman and Darvell 1990: 58, pl. 6B (Hong Kong); Wells and Bryce 1993: 117, fig. 144 (western Australia); Yonow 2001: 11, pl. 1 fig. 1 (Indonesia); Hervé 2010: 233 (New Caledonia).

#### Material.

Rosalie Moller wreck, near Hurghada, Egyptian Red Sea, 28 Apr 2015, 40 m depth, 50–60 mm alive, 21 × 10 mm preserved leg. and photographs S Kahlbrock (SK # 3).

#### Description.

**Plate 6.**
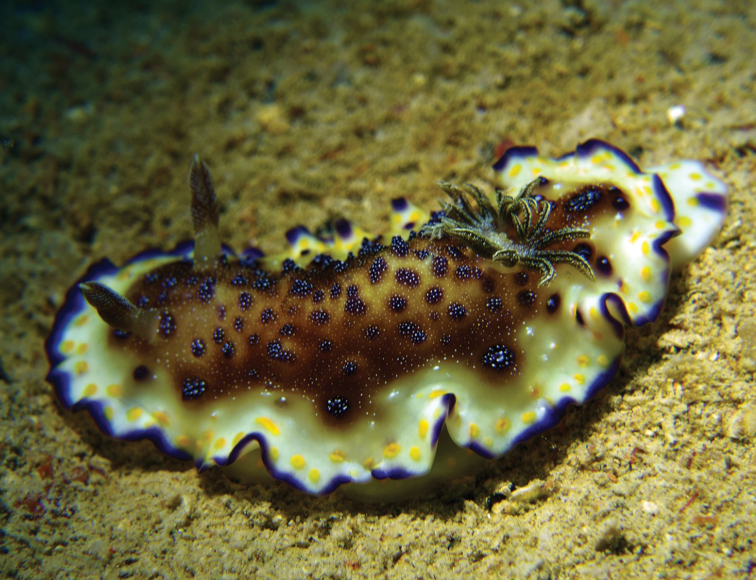
*Goniobranchus collingwoodi* (Rudman, 1987), photograph S Kahlbrock.

The photographs perfectly fit the description of this species by Rudman (1987) (Plate 6). The dorsum is cream with a purple margin. Inside the purple margin is a broad opaque white band containing many yellow spots on the outside and very few purple spots on the inside. There is an ochre zone linking the white band to the dorsal hump, which is covered in brown patches and purple spots, and both are overlain with white spots. The rhinophores are brown with the edges of the lamellae being white. The quadrangular gills have a more complicated colour pattern, translucent with a dark brown or grey line down the edges of the pinnules on both their outer and inner sides, the pigmentation extending onto both sides of the pinnules. Some of the gills are branched or forked, and they are numerous, arranged in a double spiral around the anal papilla. The foot extends a short distance behind the mantle and is white with many round yellow spots and a purple patch on the margin.

**Figure 3.**
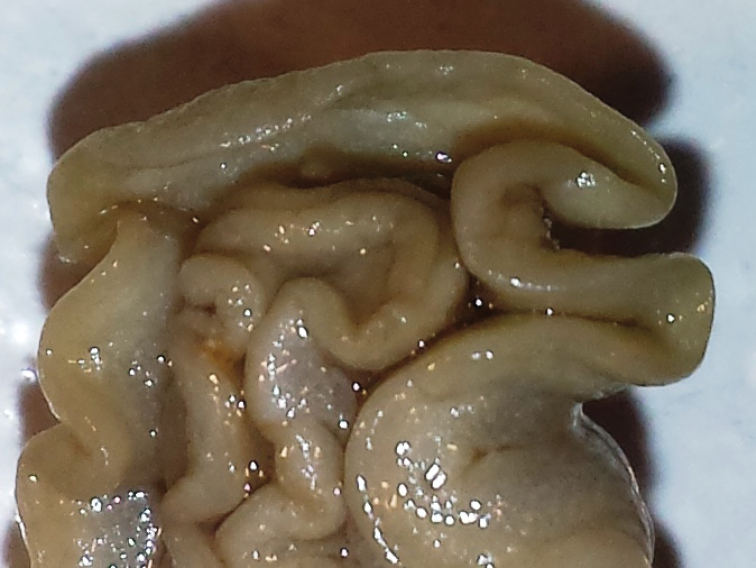
*Goniobranchus collingwoodi* (Rudman, 1987) ventral view of anterior showing head, oral tentacles, and foot margin.

Ventrally, the preserved specimen is monochromatic. The margins of the mantle and foot are contracted and the anterior margin of the foot is bilaminate (Figure 3).

#### Remarks.

This is the first record of this well-known western Pacific species from the Red Sea. There are no literature records of this species from the Indian Ocean (e.g., Tibiriçá et al. 2017, SeaSlugForum, http://seaslugs.free.fr/nudibranche/a_intro.htm). It remains to be seen if this is a one-off introduction or whether the species will establish itself in the either the Red Sea and/or the western Indian Ocean.

### Goniobranchus pseudodecorus sp. n.

Taxon classificationAnimaliaNudibranchiaChromodorididae

http://zoobank.org/228548FD-C48D-4CB8-A63F-018E6B72D1A8

Figure 4 , Plate 7

Chromodoris maculosa – Eliot 1908: 108–109 (the beacon, Khor Dongola, Suakin, Sudan) (**non**Chromodoris maculosa Pease, 1871).
Chromodoris cf. decora Yonow, 1989: 294, pl. 4 (Creek, Jeddah, Saudi Arabia, Red Sea); Perrone and Doneddu 2001: 121–130, pl. 1 figs C, D (Naama Bay, Sharm el Sheikh, Egypt, Red Sea).
Glossodoris sp. 10 Debelius & Kuiter, 2007: 149 (Eilat, Israel, Red Sea).
Chromodoris sp. Yonow, 2008: 60, 186 (Jeddah, Eilat, Red Sea).

#### Type material.

**HOLOTYPE SMF 349570**: Hotel Zabargad, 120 km south of Marsa Alam, Egypt, Feb 2003, 16 mm alive (9 × 4 mm preserved), leg. and photographs J Hinterkircher. **PARATYPE SMF 349571**: Balena wreck, Hurghada, Egypt, 02 Aug 2012, 9 m depth, approx. 15 mm alive (10 × 3 mm preserved), leg. S Kahlbrock (SK # 19).

#### Other material.

Quseir, Egypt, July 2000, approx. 10 mm alive (6 × 2.5 mm preserved), leg. and photographs J Hinterkircher (jaw and radular preparations); Jeddah, Saudi Arabia, photographs only from 1980’s, Pam Kemp, J Kuchinke, G Smith; Eilat, Israel, 18 Feb 2005, O Ledermann; near Hurghada, Egypt, 07 July 2012, 12 June 2016, S Kahlbrock.

#### Diagnosis.

Body shape rounded oblong anteriorly and rounded posteriorly. Opaque white pointed tail always longer than mantle. Dorsum translucent rose centrally and whiter marginally, with meandering longitudinal opaque white lines and round rose spots. Margin translucent orange with elongated opaque white patches.

#### Description.

**Plate 7.**
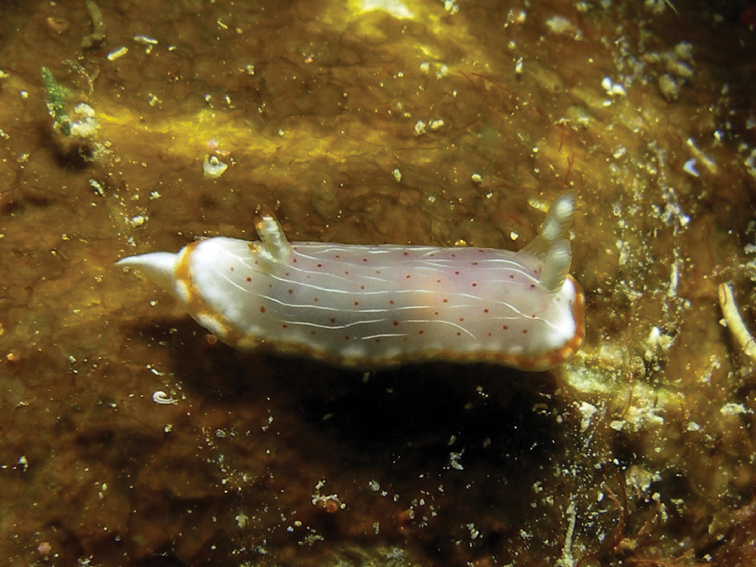
*Goniobranchus pseudodecorus* sp. n., photograph S Kahlbrock (non-type).

The shape of this species is very distinctive: all photographs depict an elongated oval body of which the anterior margin is oblong and the posterior end is rounded (Plate 7). The pointed tail extends beyond the mantle, and is translucent white with an opaque white triangular marking centrally. The mantle is translucent rose with longitudinal interrupted opaque white lines and round pink spots that are ocellated with deeper rose. Around this area is a band of white patches that may be confluent, followed by a translucent orange band containing discrete white patches. The rhinophores are translucent orange with two inner opaque white areas; there are up to 12 lamellae and the translucent stalks issue from translucent, slightly raised sheaths. The 6–8 unipinnate gills are arranged in a simple circle; they are also translucent with opaque white cores; the orange pigment in the tips is within the translucent area.

**Figure 4.**
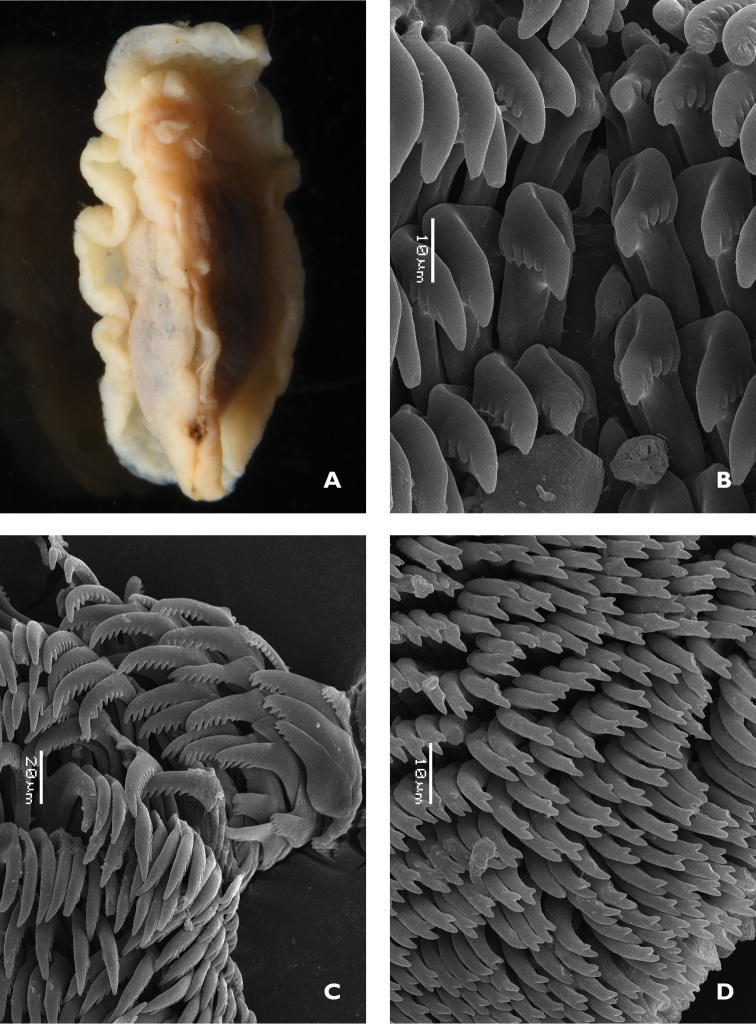
*Goniobranchus pseudodecorus* sp. n. **A** ventral view of anterior showing head, oral tentacles, and foot margin **B** midline area from the anterior portion of the radula **C** lateral teeth from the anterior section of the radula **D** jaw rodlets.

The preserved specimens are not totally contracted, and still retain the opaque white lines on the dorsum; however, no coloured spots remain on any of the specimens. The almost black digestive gland within is clearly visible. The edges of the foot are slightly crumpled, squared anteriorly, and the oral tentacles are visible as swollen nipples (Figure 4A). The anterior margin of the foot does not appear to be bilaminate. The 2012 specimen from Hurghada is aberrant in having two left rhinophores.

The notes made on the paratype on arrival read as follows: “dorsum dense, opaque dirty orange, glistening white lines, coloured areas still visible on rhinophores and gills (the latter were darker). Two left rhinophores but one right. Mantle margin distinct, separate, mantle glands visible posteriorly. Ventrally, the hyponotum a darker orange, foot lighter. Foot anterior margin angular with a slight median dent, large swollen oral tentacles.”

The reproductive system is developed in the 6 mm specimen (collected in the summer), despite its being smaller than the types and the average recorded length, with ducts and glands clearly visible as well as the bursa copulatrix.

This same specimen has a radular formula of 27–28 × 28–33.1.33–28. There is a small (up to 15 µm long) central triangular tooth medially, crowded by the first lateral teeth (Figure 4B). The first lateral is twisted on itself, with one or two large denticles medially and a row of four small saw-like denticles laterally (Figure 4B). The length of the cusp increases quickly to approximately tooth 9 as does the number of denticles, also to nine. In this region, the twist of the cusp is still pronounced and forms a small knob at the top of the root/cusp junction. At approximately tooth number 13–15 until the last five teeth, the cusps are somewhat straighter on the root with a pronounced knob on the top (Figure 4C); the denticles are saw-like. The last five teeth are stacked together and very reduced in size, flattened plates tapering towards the end with few denticles (Figure 4C).

The jaws are composed of curved rodlets. These are bifid on the tip, with one denticle being much smaller than the other (Figure 4D).

#### Remarks.

Although Eliot (1908) compares his specimen to *Chromodoris maculosa* Pease, subsequent records have shown it to be quite different and consistently so over time. *Goniobranchus pseudodecorus* sp. n. has been recorded from the Red Sea a number of times (Yonow 1989, Perrone and Doneddu 2001, http://www.seaslugforum.net/find/13213, Yonow 2008). *Goniobranchus decorus* (Pease), to which this species has been compared, does not occur in the Red Sea but has a western Pacific distribution: it is similar to *G. pseudodecorus* sp. n. in having a translucent orange margin and pointed white foot, but there are different white markings on the dorsum, large purple patches in the orange and white marginal bands, and the rhinophore pigmentation is without banding. The body is not so obviously angular anteriorly as in *G. pseudodecorus* sp. n. The radular formula of a 16 mm-preserved specimen from Australia is 52 (+3) × 48.0.48 and the teeth vary similarly along the row (Rudman 1986b: 331).

#### Distribution.

Endemic to the Red Sea. The first record of this species is by Eliot (1908) from the Sudanese Red Sea in which he describes the same obvious characters of shape and colour: “Elongated and rather flat: mantle broad, especially over head. Foot ends in sharp point projecting… Gills small and thick, seven in number, simply pinnate, the two hindmost smaller. … Colour translucent greyish pink. … broad undefined band of opaque white, and outside, bordering the mantle, a broad transparent orange-yellow line interrupted by opaque white spots along the edge.” The species is clearly endemic to the Red Sea, and I suspect that the Maldives locality of the second photograph in Debelius and Kuiter (2008) is erroneous.

#### Derivatio nominis.

An unimaginative name alluding to the similarities with *Goniobranchus decorus*.

### Hypselodoris dollfusi

Taxon classificationAnimaliaNudibranchiaChromodorididae

(Pruvot-Fol, 1933)

Figure 5 , Plate 8

Glossodoris dollfusi Pruvot-Fol, 1933: 126, pl. I figs 7, 8; fig. 40 (Red Sea).
Hypselodoris dollfusi – Gosliner and Behrens 2000: 116, Figs 1B, 4, 5 (Oman); Yonow 2008: 60, 192 (Red Sea).

#### Material.

Wreck of ‘Rosalie Moller’, near Hurghada, Egypt, 01 Aug 2012, 33 m depth, one specimen approx. 50 mm (approx. 25 × 15 mm preserved, curled), leg. and photographs S Kahlbrock.

#### Description.

**Plate 8.**
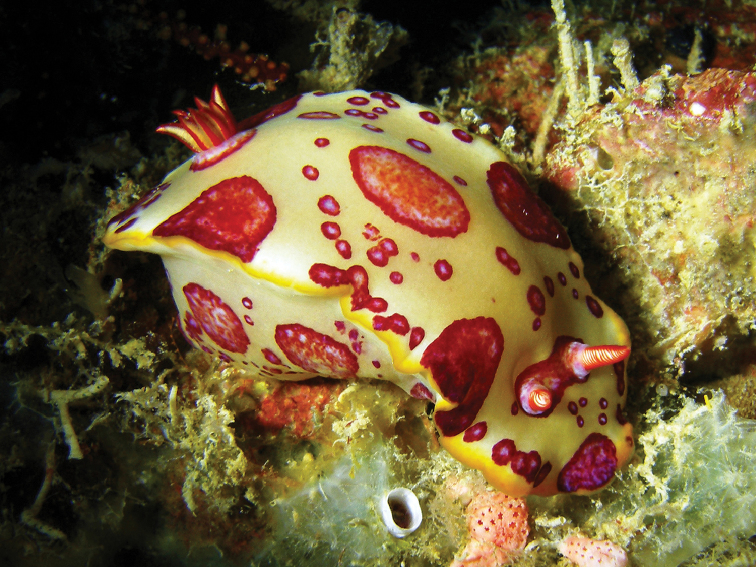
*Hypselodoris dollfusi* (Pruvot-Fol, 1933), photograph S Kahlbrock.

This specimen represents the first and nearest record to its type locality for a species originally described from the Red Sea 80 years ago, and is thereby removed from its *incertae sedis* status of Yonow (1989). It is clearly distinct and recognisable from all the Red Sea chromodorids: the body is very large, firm, and with a high profile. It is pale to dark yellow with series of large and small spots, which can be shades of red and pink, often with a red margin, and a yellow margin encircling the mantle (Plate 8).

**Figure 5.**
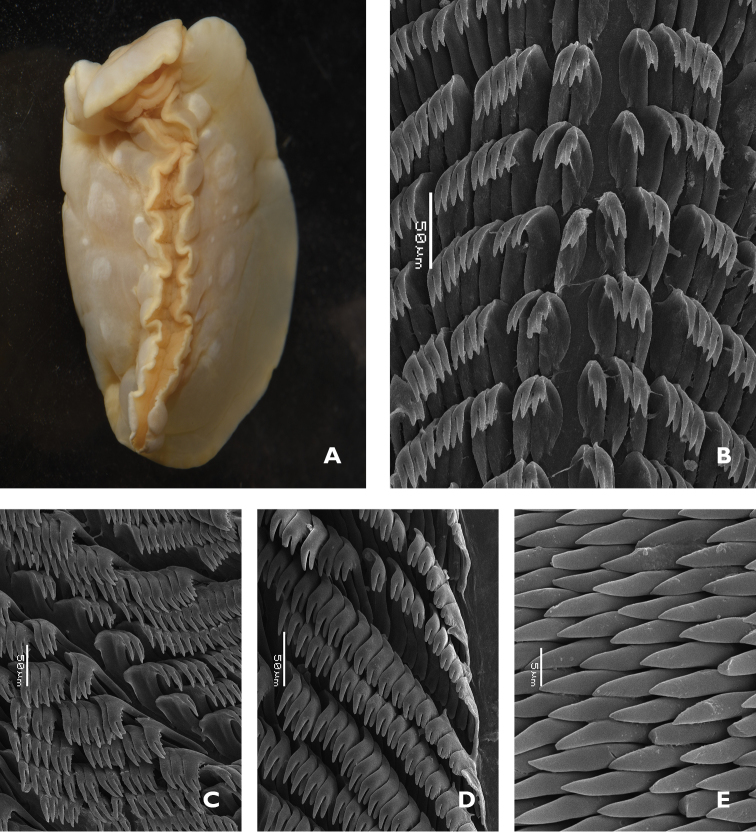
*Hypselodoris dollfusi* (Pruvot-Fol, 1933) **A** ventral view of the whole specimen showing head, oral tentacles, and deep hyponotum with raised spots **B** midline area from the anterior portion of the radula **C** lateral teeth from the posterior portion of the radula **D** lateral teeth from the middle section of the radula **E** jaw rodlets.

The preserved specimen is beige (examined 2013) with an orange margin. The patches and spots remain visible as red or faded red. There are red spots also present on the gill pocket, on the gills, around the margin of the hyponotum (large), and on the top of the foot (small, fading). The gonopore is surrounded by a red ring. The rhinophore pockets are white and retain their red margins. The mantle glands are visible as a series of darker yellow patches at the very posterior of the margin (Figure 5A).

Ventrally, the body is swollen, cream-coloured, and the red spots visible as opaque white slightly raised spots (Figure 5A). The head is rounded, the tentacles just visible. The anterior margin of the foot is very angular, bilaminate, with both laminae notched.

The reproductive system of the single specimen preserved in the summer is well developed.

Its radular formula is > 65 × 71.0.71. There is a clear space in the middle of the complete length of the radula. The first laterals on each side are identical and asymmetrical: all the teeth are clearly bicuspid but the first lateral has an additional small sharp cusp on its inner face (Figure 5B). The remaining laterals are typically hypselodorid and regular along the row. The last 15 or so teeth become rapidly reduced in size with the addition of a line of 3–5 denticles on the lower cusp (Figure 5C). Along the posterior portion of the radula, from approximately tooth 30 (if not earlier) the teeth are secondarily denticulate (Figure 5D).

The jaws of *Hypselodoris dollfusi* are simple pointed rods with a slight curve (Figure 5E).

#### Remarks.

It is remarkable that this species was described in 1933 and then not seen again until 1999. Gosliner and Behrens (2000) rediscovered the species based on specimens from the Persian Gulf. Although there is only one specimen from the Red Sea, there have been numerous photographic records from the northern gulfs of Eilat and Suez since Yonow (2008). It has since been photographed with some regularity but appears to be more common in the Persian Gulf (http://www.seaslugforum.net/find/hypsdoll, https://www.facebook.com/pg/uaebranchers/photos/?ref=page_internal).

White (1951) described a specimen from the Bannwarth Collection (NHM) as “…resembling a young individual of *Glossodoris luteorosea*…”. While she does not describe any remaining pigment, the only species in the Red Sea with large red spots similar to the Mediterranean species is *H. dollfusi*. However, White describes nine gills (while there are ten in Gosliner and Behrens (2000) and eight in this specimen; one is bifurcated) and “… cream coloured, soft and semi-transparent. The mantle edge is undulating.” This does not agree at all with the preserved specimen of *H. dollfusi* described here. Additionally, she describes a radular formula of 54 × 30.0.30 in an 11 mm-long preserved specimen (66 × 88.0.88 in a non-measured specimen in Gosliner and Behrens 2000), and > 65 × 71.0.71 in this 25 mm-long preserved specimen. Not only does the formula differ considerably, but also the form of the teeth differs in that the first lateral has two denticles and the second lateral has one denticle.

#### Distribution.

The species is known only from the northern Red Sea (Yonow 2008), the Persian Gulf (Glayzer et al. 1984, Dipper and Woodward 1989, Gosliner et al. 2008), and the Gulf of Oman (Gosliner and Behrens 2000).

### Miamira magnifica

Taxon classificationAnimaliaNudibranchiaChromodorididae

Eliot, 1910

Figure 6 , Plate 9

Miamira magnifica Eliot, 1910: 432, pl. 25 figs 10, 11 (Seychelles); Yonow 1994: 123 (Maldive Islands); Yonow 2008: 61, 206 (Red Sea); Tibiriçá et al. 2017: 40, fig. 11G, H (Mozambique).

#### Material.

Marine Biological Laboratory, Eilat, Israel, 09 Aug 1983, 10 m depth, one specimen 31 × 16 mm (preserved), leg. and photographs J Dafni.

#### Description/remarks.

There is so much confusion surrounding this species that the Red Sea specimen is here described and illustrated in detail to enable clear recognition. As succinctly stated by Rudman (2007), a review of the genus by Valdés and Gosliner (1999) which synonymised several genera actually omitted two crucial species, and so the confusion continues. In concurrence with Rudman, *Miamira magnifica* is here reported as having an Indian Ocean distribution, including the Red Sea (also in Yonow 2008: 206). This is a correction of Yonow (1994) who stated that it had an Indo-West Pacific distribution because *flavicostata* from Australia and Japan had been included as a possible synonym.

**Plate 9.**
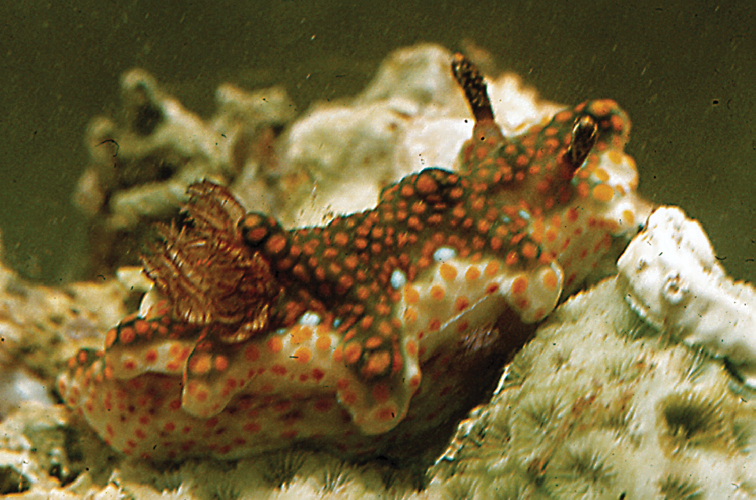
*Miamira magnifica* Eliot, 1910, photograph J Dafni.

Despite much searching, this remains the only specimen record of *Miamira* from the Red Sea. The specimen was examined and drawn by the author when it was moribund: it was pale green with white nodules, each of which were encircled by two or three blue rings (Plate 9); the central two nodules were the largest. The shape of this central green area was like a cross of Lorraine, a longitudinal central line with two crossbars. Outside this region, the mantle was white with raised orange spots, which also were present on the slightly raised tubercles covering the sides and white hyponotum of the specimen. The demarcation between the mantle and the sides was clearly marked by orange dots. The shape of the mantle was very regular and its texture firm, the foot extended beyond it.

**Figure 6.**
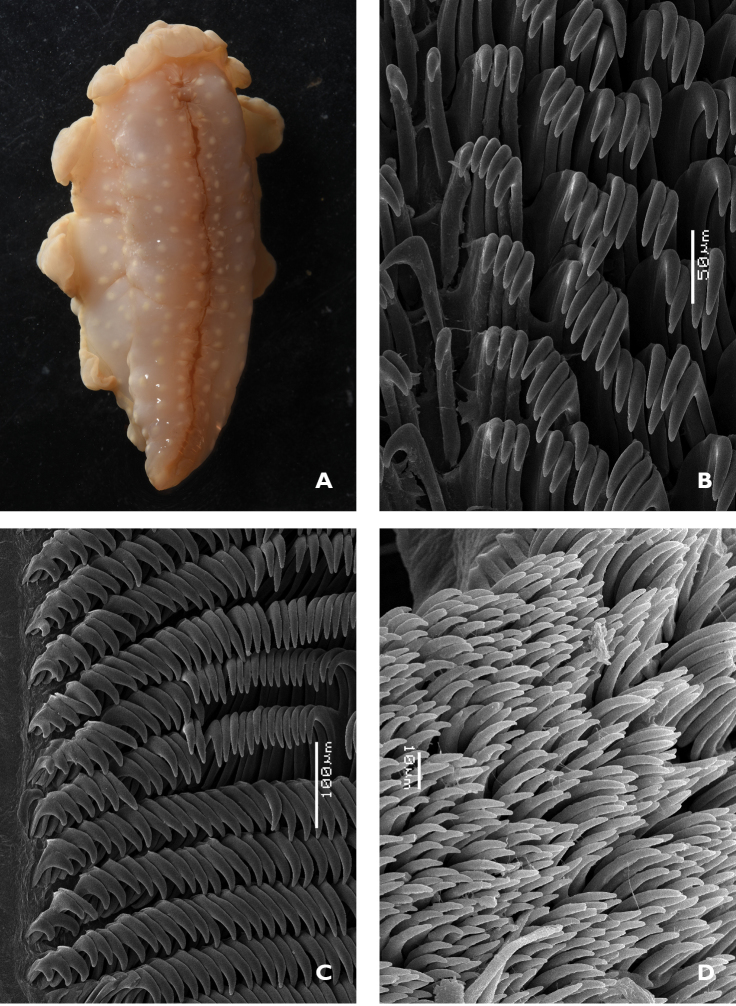
*Miamira magnifica* Eliot, 1910 **A** ventral view of whole specimen **B** midline area from the anterior portion of the radula **C** lateral teeth from the anterior section of the radula **D** jaw rodlets.

The preserved specimen retains much of the original shape, albeit somewhat contracted, and the spots are clearly visible (Figure 6A). The reproductive system of the single specimen was developed when it was collected in the summer.

The radula comprises at least 80 rows of simply hooked teeth; there are approximately 100 teeth in a row. There is no rhachidian and the last few teeth in each row are greatly reduced in size and stacked together (Figure 6B, C). This compares well with those of a Maldives specimen measuring a very similar 30 mm having a radular formula of 102 × approx. 90.0.90 (Yonow 1994).

The jaws are simple rodlets with pointed tips and a slight curve (Figure 6D).

#### Distribution.

Northern Red Sea, tropical western Indian Ocean (Yonow 1994, Tibiriçá et al. 2017, http://www.seaslugforum.net/showall/ceramagn).

### Verconia sudanica

Taxon classificationAnimaliaNudibranchiaChromodorididae

(Rudman, 1985)

Plate 10

Noumea sudanica Rudman, 1985: 254, figs 1e, 7b, 8 (Red Sea); Yonow 2008: 61, 200 (Red Sea).

#### Material.

South of Hurghada, Egypt, 22 Jan 2009, 2–4 m depth on rocks during night dive, four specimens 15–20 mm alive approx. (6.5, 7, 7, 8 mm preserved); leg. and photographs S Kahlbrock; photographs of numerous individuals, the Creek, Jeddah, Saudi Arabia, 1970–1994, W Pridgen, D & S Sharabati; photographs only, vicinity of Hurghada, Egypt, 13 Aug 2012, S Kahlbrock; photographs of the type specimen, Suakin, Sudan, 25 April 1980, leg. and photographs C Todd (Australian Museum C.131570).

#### Description.

**Plate 10.**
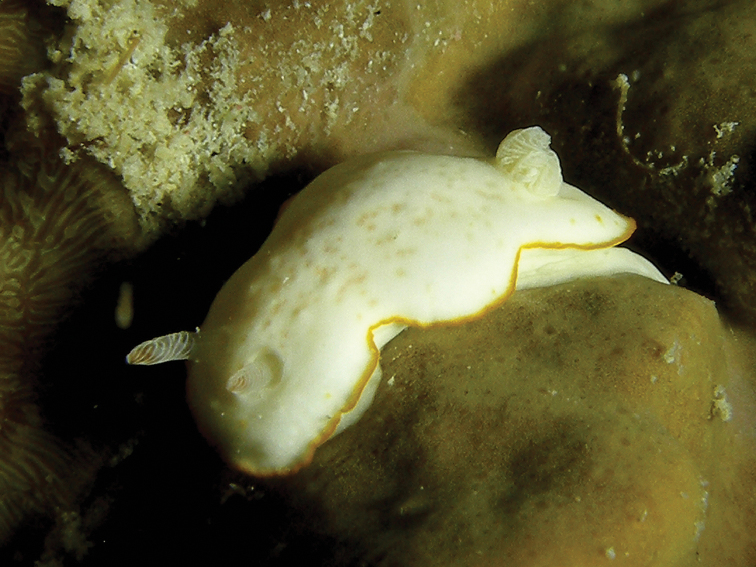
*Verconia sudanica* Rudman, 1985, photograph S Kahlbrock.

Since Rudman’s (1985) and Yonow’s (2008) records, *Verconia sudanica* has been recorded somewhat more frequently in the Red Sea (http://www.seaslugforum.net/showall/noumsuda, https://www.inaturalist.org/taxa/126112-Noumea-sudanica). It is described here from specimens collected in the northern Red Sea. The excellent series of photographs depict five individuals, of which three are grouped together (four specimens were collected). In the photograph of the group, the rhinophores and nine plumose but simply pinnate gills are all pure white. The edges of the rhinophore lamellae (numbering 9–11) and main axes of the gills (facing outwards) are opaque white. In photographs of a single animal, the apical one third of the rhinophore is very faintly orange, with opaque white edges to the 9–11 lamellae. All five animals have a white dorsum with pits and a narrow yellow margin, which is faintly darker orange at the edge (Plate 10). In two individuals, the pits are slightly yellow and in a third one, there are small conical papillae dotted on the surface. The foot is also white, and the tip is bordered with a yellow line; one photograph from the side shows that the margins of the foot are white.

The preserved specimens are identical, opaque cream with a thickened but slightly lighter coloured margin containing single round mantle glands that are semi-translucent. The gill pocket is large and slightly raised and in two specimens, the unipinnate gills barely protrude. The foot extends beyond the mantle slightly in all but one specimen. The foot is angular anteriorly, and the large oral tentacles are clearly visible.

#### Remarks.

There are no similar species in the Red Sea; only the western Pacific *Verconia simplex* (Pease, 1871) is equally small, white to pale pink, with a bright orange margin; however, there are no specimen records further to the two photographs in Yonow (2008) so these records remain unconfirmed. *Verconia simplex* has bright orange tips to both the rhinophores and the gills.

Some comments are necessary on the generic placement of this species. Gosliner and Johnson (2012: 6) found that *Noumea* was not monophyletic but its species were distributed in two clades (and within other genera): “*Noumea* consists of two separate clades (both pp = 1.00) that are poorly supported as a combined clade in the analysis when variable positions are included (pp = 0.61). Although this support is not sufficient, all of the species in both of these clades are currently named *Noumea* and will retain this name in order to maintain stability.”

With this, they synonymised *Verconia*, a monotypic genus containing *V. verconis* (Basedow & Hedley, 1905). However, they failed to recognise that *Noumea* was preoccupied. WoRMS has a small note to that effect and *Verconia* (as a synonym) should be the correct generic designation (http://www.marinespecies.org/aphia.php?p=notes&id=279974) despite its type species being morphologically different: “*Noumea* Risbec, 1928 (Mollusca: Gastropoda) is a junior homonym of *Noumea* Fauvel, 1874 (Arthropoda: Coleoptera), a name in current use. *Verconia* Pruvot-Fol, 1931 was recognized as a synonym of *Noumea* Risbec by Johnson and Gosliner (2012) and is here used for species of the chromodoridid group previously known as *Noumea*.”

Examination of Rudman’s (1984) review of the chromodorid genera and his descriptions of species of *Noumea* (Rudman 1986a, b) show that these two genera are in fact similar, but because the external morphology of *Verconia verconis* is so distinctive, it has historically been considered in its own genus. The radular formulae and teeth shapes are very similar, as are some morphological characters (although these are also similar to other chromodorid genera). The reproductive systems vary a little within the group described as *Noumea* by Rudman (1984, 1986a, b although some species belonging to a second clade have been re-assigned to *Diversidoris*) and in some cases are more similar to that of *V. verconis* (see also Rudman 1986b: 402). As it is very unlikely that the specimens of *V. verconis* used in the molecular analysis by Johnson and Gosliner (2012) were misidentified, the synonymy must be accepted, but it is unfortunate that these authors did not examine the literature and, as a result, the species they assigned to *Noumea* must now be reassigned to *Verconia*.

#### Distribution.

Endemic to the Red Sea.

## Conclusions

It is unfortunate that the photographic records of the chromodorids included in "Sea Slugs of the Red Sea" (Yonow 2008) and of *Diversidoris aurantionodulosa* in Yonow (2015) could not be substantiated by specimen collections. This paper completes the identification of all the available chromodorid specimens in the author’s collections from the Red Sea; forty-one species of chromodorids has been recorded from the Red Sea to date, but there are an additional dozen species known from the literature (see checklist in Yonow 2008) or only from photographs (Yonow 2008, 2012; pers. obs.).

In the event that some species groups, such as the ‘*Glossodoris cincta*’ group, need further work, all available specimens will have been identified, described, and lodged in the Senckenberg Museum. The appendix lists material of six species commonly found in the Red Sea and recorded previously, and these are also deposited in the Museum.

While the length of time taken to publish some of these records has been substantial, one benefit has been that a vast number of photographs from various sources could be analysed to trace and date first records of ‘new’ species records. Hence, while the newly described species have been present in the Red Sea for the last four or five decades at least, *G. collingwoodi* and *D. flava* are almost certainly more recent introductions. I suspect it will be with the aid of ‘citizen scientists’ that more records of these species, and their establishment or not within the Red Sea, or at least the northern part of it, will be made.

## Supplementary Material

XML Treatment for Chromodoris strigata

XML Treatment for Diversidoris flava

XML Treatment for Doriprismatica kyanomarginata

XML Treatment for Glossodoris kahlbrocki

XML Treatment for Goniobranchus collingwoodi

XML Treatment for Goniobranchus pseudodecorus

XML Treatment for Hypselodoris dollfusi

XML Treatment for Miamira magnifica

XML Treatment for Verconia sudanica

## Figures and Tables

**Plate 11.**
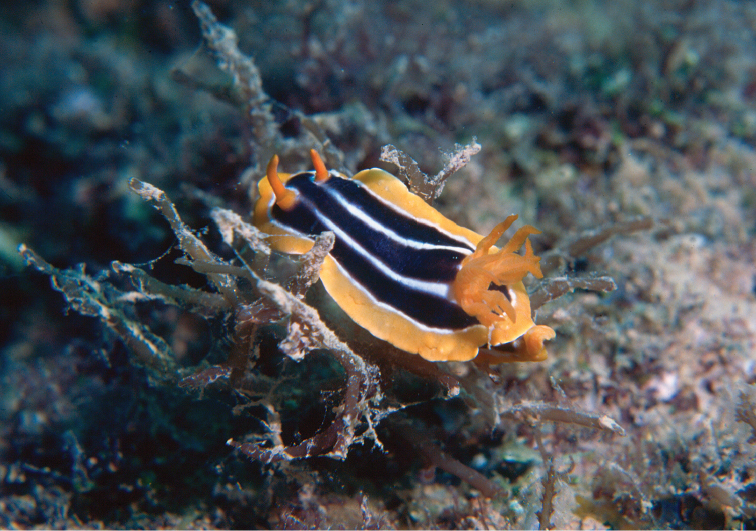
*Chromodoris africana* Eliot, 1904, photograph E Pridgen.

**Plate 12.**
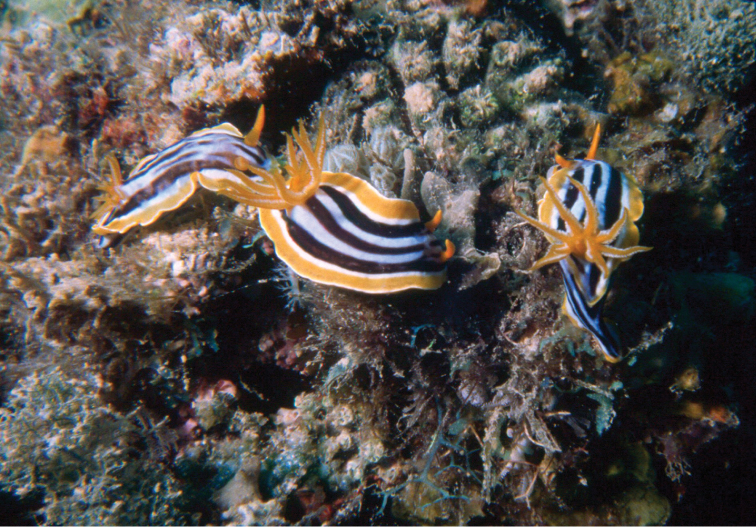
*Chromodoris quadricolor* (Rüppell & Leuckart, 1830), photograph E Pridgen.

**Plate 13.**
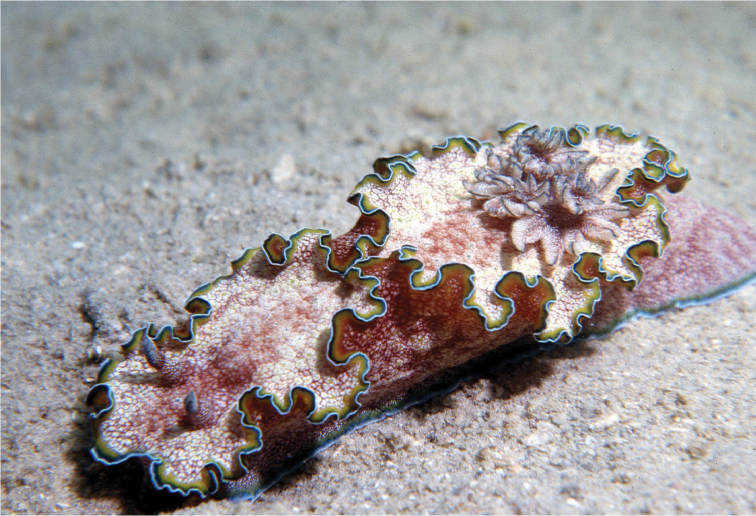
*Glossodoris cincta* (Bergh, 1888), photograph E Pridgen.

**Plate 14.**
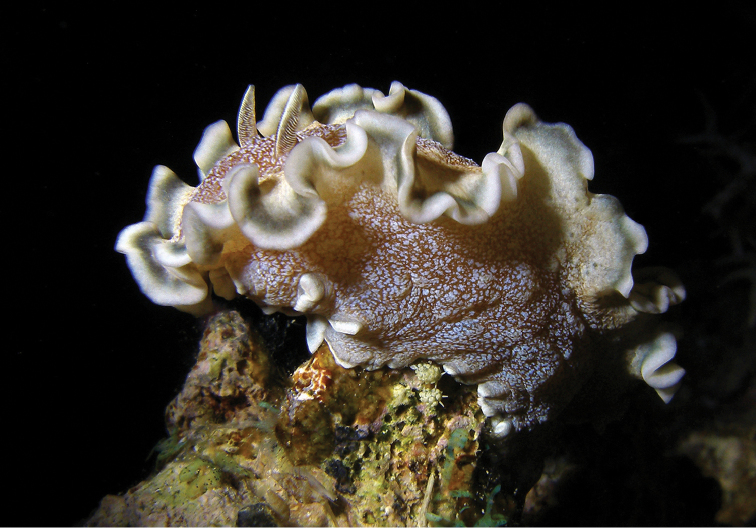
*Glossodoris hikuerensis* (Pruvot-Fol, 1954), photograph S Kahlbrock.

**Plate 15.**
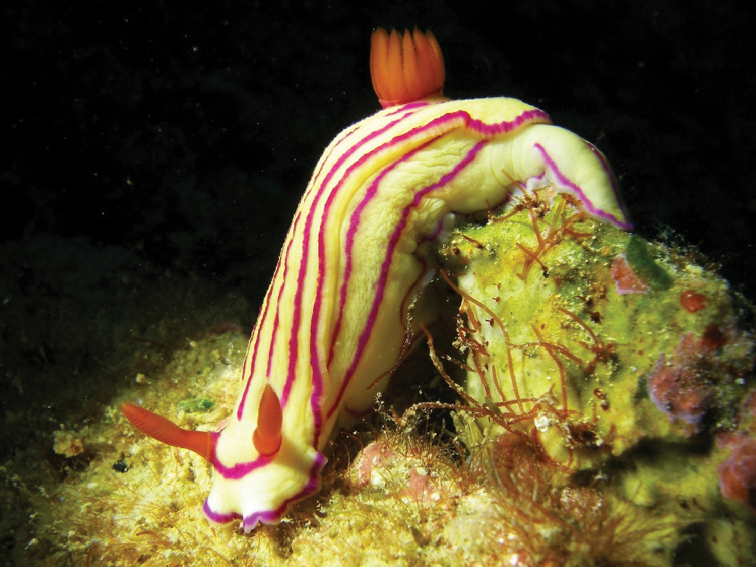
*Hypselodoris maridadilus* Rudman, 1977, photograph S Kahlbrock.

**Plate 16.**
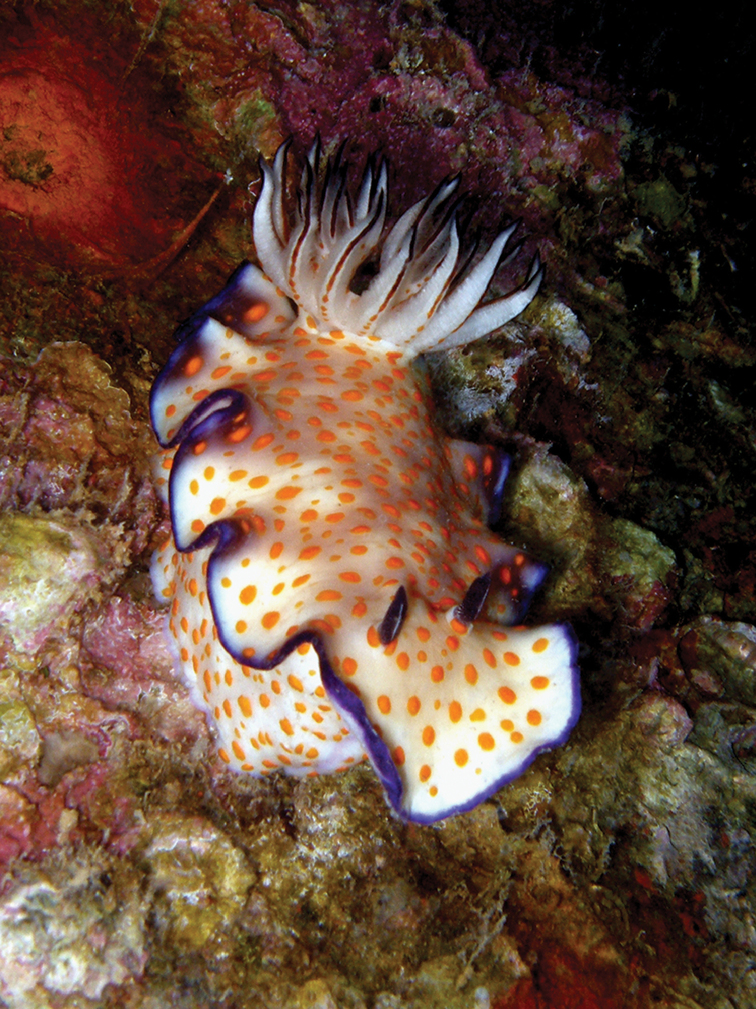
*Risbecia pulchella* (Rüppell & Leuckart, 1830), photograph S Kahlbrock.

## Appendix 1

Additional specimen and photographic records of species of Chromodorididae previously described in Yonow (1988)

***Chromodoris africana* Eliot, 1904**

• Near Garden, near Sha’arm el Sheikh, Egypt, 24 Dec 1990, 18 m depth (sandy rubble substrate with boulders), 37 × 9 mm alive, leg. and photographs N Yonow (NY # 109) [2 black lateral stripes, 2 white with faint blue tinge, broad mantle margin with white margin; orange pocket rims; fine white gill pinnules].

• Ras umm Sid, near Sha’arm el Sheikh, Egypt, 25 Dec 1990, 25 m depth (sandy rubble substrate with boulders), 40 × 8 mm alive, leg. and photographs N Yonow (NY # 113) [black laterally with 2.5 blue lines, thin stripe between rhino (does not widen like in *quadricolor*), less blue than *quadricolor*. Large gill pocket, ten gills, orange pocket rim and anal papilla].

• Wreck, Ras Mohammed, Egypt, 27 Dec 1990, 10 m depth, 62 × 10 mm alive, leg. and photographs N Yonow (NY # 117) [white margin, wide orange submargin + narrower white + wide black; broken dorsal black; 10+ gills + rhino dark orange’. Large gill pocket, orange rim also rhino pocket].

• Egypt, 24 Sept 1995, 30 m depth, two specimens 13 mm relaxed and min. 25 mm bent, both preserved (HU-02), leg. Á Valdés & E Mollo.

• Egypt, Sept/Oct 1995, one specimen min. 11 mm preserved, dissected, and dried out at some point (HU-M5), leg. Á Valdés & E Mollo.

• The creek, Jeddah, Saudi Arabia, 1970–1994, numerous photographs of numerous individuals, Pam Kemp, G Bemert, W Pridgen.

***Chromodoris quadricolor* (Rüppell & Leuckart, 1830)**

• South side Tiran Island, Ras Mohammed, 13 m depth (sandy gravelly substrate with isolated large boulders covered in epifauna), 23 Dec 1990, leg. and photographs N Yonow (NY # 110) [3 black lateral stripes, 3 blue, 13+ gills. More svelte than *africana*. Orange blur behind rhino. Orange gill + rhinophore pockets]; two individuals not collected (NY # 105, NY # 106), 17 × 5 mm and 40 × 8 mm [both perfect *C. quadricolor*].

• Near Garden, near Sha’arm el Sheikh, Egypt, 24 Dec 1990, 9 m depth (sandy rubble substrate with boulders), 25 × 5 mm, leg. N Yonow (NY # 114) [blurry, three black strips laterally].

• Ras umm Sid, near Sha’arm el Sheikh, Egypt, 25 Dec 1990, 25 m depth (sandy rubble substrate with boulders), 12 × 7 mm alive, leg. and photographs N Yonow (NY # 115).

• The creek, Jeddah, Saudi Arabia, 1970–1994, numerous photographs of numerous individuals, Pam Kemp, G Bemert, W Pridgen, N Yonow.

• Egyptian Red Sea, 1995–2001, numerous photographs of numerous individuals, J Hinterkircher.

***Glossodoris cincta* (Bergh, 1888)**

• Jeddah, Saudi Arabia, 1970–1980, photographs of three individuals, P Kemp.

• The creek, Jeddah, Saudi Arabia, 1970–1994, numerous photographs of numerous individuals, W Pridgen [common], Plate 13.

• Egyptian Red Sea, 1995–2001, numerous photographs of numerous individuals, J Hinterkircher; 2009–2016, S Kahlbrock.

All photographs depicting individuals with the wide yellow-ochre submarginal band, blue-black marginal line, and the very edge marked in white.

***Glossodoris hikuerensis* (Pruvot-Fol, 1954)**

• Egypt, 29 Sept 1995, 25 m depth, photograph of one individual (HU-023), Á Valdés & E Mollo.

• Marsa Alam, Egypt, Feb/March 2001, photographs of one individual, J Hinterkircher; 2009–2016, S Kahlbrock. Plate 14.

***Hypselodoris maridadilus* Rudman, 1977**

• Egypt, 26 Sept 1995, intertidal, one specimen 14 × 7 mm preserved (HU-019), leg. Á Valdés & E Mollo.

• Jeddah, Saudi Arabia, 1970–1980, photographs of three individuals, P Kemp.

• The creek, Jeddah, Saudi Arabia, 1970–1994, photographs of two individuals, W Pridgen.

• Egyptian Red Sea, summer 1995, photographs of one individual, J Hinterkircher; 2009–2016, numerous photographs of several individuals, S Kahlbrock. Plate 15.

***Risbecia pulchella* (Rüppell & Leuckart, 1830)**

• Ufornakes reef, 20 km s of Hurghada, Egypt, 15 Aug 1965, one specimen 18 mm long × 13 mm high preserved, leg. Linsenmair [like T Paulus MSS # 7 but with slightly raised spots like SK # 5].

• Wreck, Aqaba, Jordan, Mar 1990, one specimen 37 mm long × 13 mm high preserved (MSS # 7), leg. and photo T Paulus [no markings or spots cf. recent specimen S Kahlbrock # 5].

• Egypt, 24 Sept 1995, one specimen 27 × 7 mm preserved (HU-01), leg. and photograph Á Valdés & E Mollo [had dried out in the past, brittle].

• MS Balena, Hurghada, Egypt, Apr 2015, 9 m depth, two specimens 48 mm long × 23 mm high and 46 mm long × 22 m high preserved (SK # 5), leg. and photographs S Kahlbrock [opaque raised spots].

• The creek, Jeddah, Saudi Arabia, 1970–1994, numerous photographs of numerous individuals, many in mating pairs, W Pridgen.

• Egypt, 1995–2001, numerous photographs of numerous individuals, J Hinterkircher; 2009–2016, S Kahlbrock. Plate 16.
